# High-energy-density dual-ion battery for stationary storage of electricity using concentrated potassium fluorosulfonylimide

**DOI:** 10.1038/s41467-018-06923-6

**Published:** 2018-10-26

**Authors:** Kostiantyn V. Kravchyk, Preeti Bhauriyal, Laura Piveteau, Christoph P. Guntlin, Biswarup Pathak, Maksym V. Kovalenko

**Affiliations:** 1 Laboratory of Inorganic Chemistry, Department of Chemistry and Applied Biosciences, ETH Zürich, Vladimir-Prelog-Weg 1, CH-8093 Zürich, Switzerland; 2 Laboratory for Thin Films and Photovoltaics, Empa—Swiss Federal Laboratories for Materials Science and Technology, Überlandstrasse 129, CH-8600 Dübendorf, Switzerland; 3 Discipline of Chemistry, School of Basic Sciences and Discipline of Metallurgy Engineering and Materials Science, Indian Institute of Technology (IIT) Indore, Indore, Madhya Pradesh 453552 India

## Abstract

Graphite dual-ion batteries represent a potential battery concept for large-scale stationary storage of electricity, especially when constructed free of lithium and other chemical elements with limited natural reserves. Owing to their non-rocking-chair operation mechanism, however, the practical deployment of graphite dual-ion batteries is inherently limited by the need for large quantities of electrolyte solutions as reservoirs of all ions that are needed for complete charge and discharge of the electrodes. Thus far, lithium-free graphite dual-ion batteries have employed moderately concentrated electrolyte solutions (0.3–1 M), resulting in rather low cell-level energy densities of 20–70 Wh kg^−1^. In this work, we present a lithium-free graphite dual-ion battery utilizing a highly concentrated electrolyte solution of 5 M potassium bis(fluorosulfonyl)imide in alkyl carbonates. The resultant battery offers an energy density of 207 Wh kg^−1^, along with a high energy efficiency of 89% and an average discharge voltage of 4.7 V.

## Abstract (web summary)

Lithium-free graphite dual-ion battery offers a new means of energy storage. Here the authors show such device utilizing a highly concentrated electrolyte solution of KFSI in alkyl carbonates that exhibits a high energy density and high energy efficiency as well as an average discharge voltage of 4.7 V.

## Introduction

The worldwide increase in energy consumption and the intermittent nature of renewable sources (e.g. solar and wind energy) require grid-connected stationary energy storage systems (ESS) with higher capacities. The International Electrotechnical Commission reported that within the next 30 years, the current available capacity of ESS (ca. 141 GW) needs to be doubled or tripled. In this context, large and inexpensive stationary rechargeable batteries should be used in combination with the more traditional pumped hydroelectric storage (PHS), which arguably has reached or is approaching its maximum usable capacity^1^. However, the use of rechargeable Li-ion batteries on a gigawatt scale is widely considered problematic, especially considering the limited amount and non-uniform distribution of natural lithium reserves. Hence, there is an urgent need for new large-scale battery technologies that only use inexpensive components and naturally abundant elements. Thus far, these requirements have been fully met only by the PHS (which constitutes 99.3% of the global ESS), which has a low capital cost (0.1–1.4 compared to 15–100 ¢/kWh-cycle for Li-ion batteries). However, besides its arguably lowest energy density (0.5–1.5 compared to 75–200 Wh kg^–1^ for Li-ion batteries)^2^, PHS also suffers from geographic limitations because of the needs in terms of water and elevation.

The last two decades have seen a surge of reports on Li-ion-free batteries. For instance, other single and multivalent cations are being utilized in place of Li-ions in the conventional rocking-chair-type battery concept as Na^3–6^, K^9^, Mg^7^, and Al-ion batteries^8^. Further to this, other concepts, commonly referred to as hybrid or dual-ion batteries (DIBs) attract increasing attention, wherein electroactive (inserted) ionic species are based on abundant metals (K^9–15^, Na^16–22^, Mg^23^, Ca^24^, and Al^25–33^). The major challenge with such non-rocking-chair batteries is that they lag behind Li-ion batteries in theoretical cell-level energy density. As described below in details, we estimate the limit for known anode–electrolyte–cathode combinations to be ca. 70 Wh kg^−1^ at the cell level, which is only about 1/5 that of state-of-the-art Li-ion batteries (ca. 380 Wh kg^−1^/1160 Wh L^−1^ for LiCoO_2_, ca. 410 Wh kg^−1^/1190 Wh L^−1^ for LiNi_1/3_Co_1/3_Mn_1/3_O_2_, and ca. 370 Wh kg^−1^/1080 Wh L^−1^ for LiFePO_4_; all using graphite anode). The low-energy density of these emerging batteries is rooted in their non-rocking-chair operation principle, as the large mass of electrolyte, being a reservoir of all ions needed for the operation of electrodes, has to be factored into the energy density calculation. In comparison, only a minimal amount of electrolyte is needed in rocking-chair type metal-ion batteries, where its only purpose is to establish ionic connection between the electrodes. Thus, the most promising avenue for increasing the energy density of DIBs is to maximize the ionic content of the electrolyte, without compromising the charge storage capacities of the electrodes and the voltage of the battery.

In this work, we present a DIB concept using a graphite cathode and potassium anode, called the graphite dual-ion battery (GDIB). This battery exhibits a cell-level energy density of 207 Wh kg^−1^, owing to the high weight content of the electroactive species (65 wt%) in the electrolyte [5 M solution of potassium bis(fluorosulfonyl)imide), KFSI, in alkylcarbonates] and a high operation voltage of 4.7 V. During charging, FSI^−^ ions intercalate into graphite, as confirmed with solid-state ^19^F nuclear magnetic resonance spectroscopy (NMR), while potassium electroplates on the Al current collector. The atomic-level details of the FSI^−^ anion intercalation into graphite are not only analyzed by in situ X-ray diffraction (XRD), but also verified computationally using density functional theory (DFT) simulations to obtain better insight into the staging phenomenon of such intercalation.

## Results

### Graphite as a cathode for dual-ion batteries

Graphite is typically used as an anode material in commercial Li-ion batteries, wherein it uptakes Li-ion (up to charge storage capacity of 372 mAh g^−1^). In the context of DIB, however, this material serves as the cathode enable to be intercalated by AlCl_4_^−^, BF_4_^−^, PF_6_^−^, CF_3_SO_3_^−^^34^, bis(trifluoromethanesulfonimide) (TFSI^−^)^11,35^, fluorosulfonyl(trifluoromethanesulfonyl) imide (FTFSI^−^)^36^, bis(pentafluoroethanesulfonyl) imide (BETI^−^)^37^, and bis(fluorosulfonyl)imide (FSI^−^)^38^ anions from ionic liquids and organic electrolytes. The main factors that allow the efficient uptake of anions into graphite are a high degree of graphitization^39^, an increased area of the “non-basal plane”, and small anionic intercalants^38^. Such graphitic cathodes delivered cathodic capacities of up to 60–100 mAh g^−1^ at an average discharge voltage of 1.4–1.7 V vs. standard hydrogen electrode (SHE), i.e. 4.4–4.7 V vs. Li^+^/Li. The typical anode can be paired with cathodes including graphite, carbon, metallic Li and Na, and various conversion-type electrodes (Bi, Sn, Pb). In this study, we focus on Li-free electrode–electrolyte combinations that only include Earth-abundant elements.

### Mechanism and energy density of GDIB

GDIB works as a non-rocking-chair, i.e. dual-ion battery by utilizing the reversible intercalation of anions into the graphite cathode during charging (oxidation of graphite network). On the anode side, metal electroplating, intercalation, or alloying reactions might take place. The corresponding half-reactions during charging can be described as follows:

1 $${{{\rm{On}}\,{\rm{cathode}}}}:x{{{\rm{A}}^-}} + {\mathrm{Graphite}} \leftrightarrow {\mathrm{Graphite}}({{{\rm{A}}^ -}} )_x + xe^ -$$

2 $${{{\rm{On}}\,{\rm{anode}}}}:xe^ - + x{{{\rm{Cat}}^{{\rm{}}+}}} \leftrightarrow x{{{\rm{Cat}}^0}}\,\left( {{\mathrm{electroplating}}} \right)$$

3 $${\mathrm{or}}\,xe^ - + x{{{\rm{Cat}}^ +}} + {\mathrm{Material}} \leftrightarrow {\mathrm{Material(Cat)}}_x\, \\ \left( {{\mathrm{intercalation/alloying}}} \right)$$

where A^*−*^ is an anion (FSI^−^, TFSI^−^, PF_6_^−^, etc.), Cat^+^ is typically an alkali metal cation (Na^+^, K^+^, etc.). “Material” is an active material capable of uptaking metal atoms by, for instance, ionic intercalation (graphite and other carbonaceous materials)^9,10,40,41^ or alloying (i.e. Sn and Pb)^16^. The charging process ends when no cations or anions are left in the electrolyte, or when the graphite cathode or active material anode reaches its maximal charge storage capacity. Hence, calculation of the cell-level theoretical energy density must account for the capacity-matched quantity of the electrolyte. In contrast, in rocking-chair-type Li-ion batteries only the capacity-matched masses of the electrode materials need to be considered for the estimation of cell-level charge storage capacity.

We would like to emphasize that currently there exists no common practice for reporting cell-level energy density for various DIBs. Many reports focus exclusively on the specific charge storage capacity of individual electrodes. In our case, where potassium electroplating occurs on the anode, the cell-level capacity can be expressed as follows (see Supplementary Notes 1 and 2 for details):

4 $${\mathrm{Gravimetric}}\,C_{{\mathrm {cell}}} = \frac{{Fx[\mathrm{electrolyte}]C_{\mathrm {c}}}}{{Fx[\mathrm{electrolyte}] + C_{\mathrm {c}}\rho \cdot 10^3}}\left({{{{\rm{Ah}}\,{\rm{kg}}}}^{ - 1}} \right),$$

5 $${\mathrm{Volumetric}}\,C_{{\mathrm {cell}}} = \frac{{Fx[\mathrm{electrolyte}]C_{\mathrm {c}}\rho _{\mathrm {C}}}}{{Fx[\mathrm{electrolyte}] + C_{\mathrm {c}}\rho _{\mathrm {C}}\cdot 10^3}}\left({{{{\rm{Ah}}\,{\rm{L}}}}^{ - 1}} \right),$$

where *F* = 26.8×10^3^ mAh mol^−1^ (Faraday constant), *x* is the charge of electroactive species, [electrolyte] is the molar concentration of electrolyte in M (mol L^−1^), *C*_c_ is the specific gravimetric capacity of the graphite cathode in mAh  g^−1^, *ρ* is the density of the electrolyte in g ml^−1^, and *ρ*_C_ is the density of the bulk graphite in g ml^−1^. To estimate the energy density, the *C*_cell_ value must be multiplied by the average battery voltage, *E* = *C*_cell_*V*.

Using Eq. (4) and similar expression of gravimetric cell-level capacity reported by Dahn et al.^42^. for other variants of Li-free GDIBs with intercalation/alloying reaction on the anode side, as well as reported electrode capacities, battery voltages, and molarities of electrolytes, one can show that the cell-level energy densities of reported Li-free DIBs fall in the range of 22–68 Wh kg^−1^ (see Supplementary Fig. 1 and Table 1)^9–11,13,17,21,40,41,43^. The highest reported value of ca. 68 Wh kg^−1^ was for the GDIB utilizing 1 M NaPF_6_ in ethylene carbonate/ethyl methyl carbonate (EC/EMC) electrolyte^40,41^. It can also be demonstrated that the energy density is limited by the moderate concentrations of the electrolytes (typically about 0.3–1 M) or by the rather low battery voltages (e.g. 2 V for AlCl_3_-based batteries)^43^. We note that due to utilization of highly concentrated electrolytes in Li-based GDIBs (up to 4 M), much higher energy densities of up to 150 Wh kg^−1^ were reached^44–47^. Based on these considerations, here we focus on the search of highly concentrated, yet Li-free electrolyte formulation, maximizing the anodic capacity without compromising the graphite capacity.

### KFSI-graphite DIB with highly concentrated electrolyte

Generally, the solubility of a salt is determined by the lattice energy (the energy cost to overcome to form separate ions from the crystal lattice) and solvation energy of ions (the energy gain during solvation in the solvent, called hydration energy when the solvent is water). According to Gopal^48^, the melting temperature of alkali salts can serve as semi-quantitative indicator for their lattice energies. On the other hand, the solvation energy of ions is positively correlated with their charge density^49^. Therefore, we have surveyed the melting temperatures and ionic dimensions of complex monovalent anions that could intercalate into graphite and available as Li-free salts, such as FSI^−^, CF_3_SO_3_^−^, TFSI^−^, AlCl_4_^−^, BF_4_^−^, FTFSI^−^, ClO_4_^−^, PF_6_^−^, and BETI^−^. Among them, the FSI^−^ salts have particularly low melting points (ca. 99–103 °C for KFSI and ca. 109–114 °C for NaFSI) and, at the same time, a relatively small ionic size (ca. 5.4 Å in length), as compared to, for instance, TFSI^−^ (ca. 8.0 Å in length)^38^. For the solvents, two key considerations are the Lewis acidity (the ability to coordinate the anions) and electrochemical stability. Among variety of possible solvents we have selected ethylene carbonate/dimethyl carbonate (EC/DMC) solvent, because of its highest electrochemical stability window of up to 6 V^50^. Other polar solvents with higher Lewis acidity (e.g. dimethoxyethane, acetonitrile, and water) were unsuitable owing to their much lower oxidation or reduction stabilities. Importantly, we found that the molarity of the saturated solution of KFSI in EC/DMC at room temperature exceeds 5 M (65 wt% of KFSI). For comparison, the saturated solution of NaFSI in EC/DMC is only 1.8 M (37 wt% of NaFSI), in good agreement with its higher melting point. Saturated solutions of other non-Li salts mentioned above also had lower concentrations in the EC/DMC solvent mixture.

**Fig. 1:**
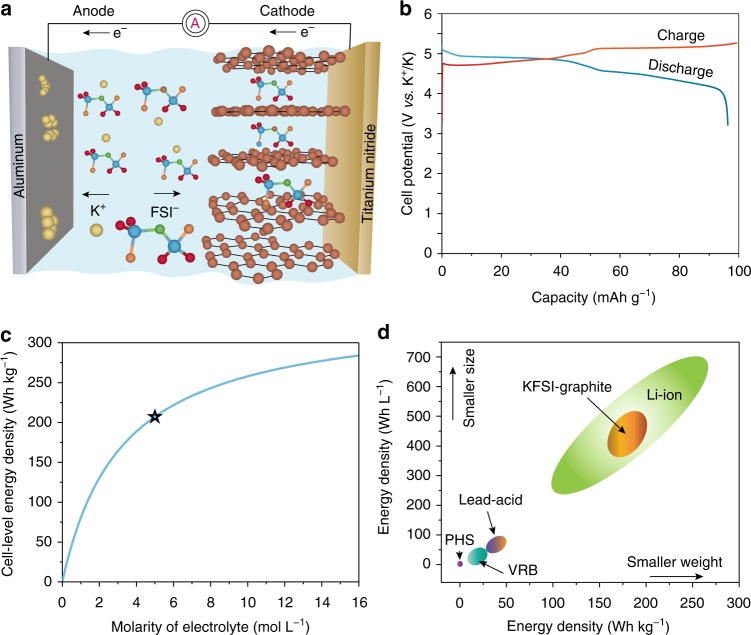
Working principle and energy density of KFSI-graphite DIB. **a** Schematic of the charging process in KFSI-graphite DIB. Fluorine, oxygen, sulfur, and nitrogen atoms in the FSI^−^ anion are shown in brown, red, blue, and green colors, respectively. **b** Typical galvanostatic voltage profile of KFSI-graphite DIB measured at a current density of 50 mA g^−1^. **c** Calculated (curve from Eq. (4)) and experimental (single point) cell-level energy density of KFSI-graphite DIB using different KFSI concentrations in EC/DMC solvent. **d** Comparison of volumetric and gravimetric energy densities of KFSI-graphite DIB battery with other battery technologies: PHS, vanadium redox battery (VRB), lead acid battery, and Li-ion battery

The mechanism of KFSI-graphite DIB can be described as follows. During charging, FSI^−^ intercalates into graphite, while K^+^ ions are reduced and deposited onto the aluminum current collector as a metallic potassium film (Fig. 1a). According to Eq. (4), the cell-level charge storage capacity will be determined by that of the graphite cathode and the molarity of the KFSI electrolyte. For energy density calculations, we assume a constant battery voltage of 4.7 V vs. K^+^/K, obtained experimentally with a 5 M KFSI/EC/DMC electrolyte (Fig. 1b). Figure 1c illustrates the capacity-limiting effect of electrolyte molarity, as the theoretical *C*_cell_ values for 1 and 5 M solutions are 17 and 44 mAh g^−1^, respectively. Finally, we obtained a high theoretical energy density of 207 Wh kg^−1^ (388 Wh L^−1^) for the KFSI-graphite DIB concept using a 5 M electrolyte, calculated from a cathodic graphite capacity of 98 mAh g^−1^ (206 Ah L^−1^).

From the perspective of grid-level energy storage, an energy density of ca. 200 Wh kg^−1^ compares favorably to established commercial technologies, such as lead-acid batteries (30–50 Wh kg^−1^) and vanadium redox-flow batteries (10–30 Wh kg^−1^). A more detailed comparison with major battery technologies that are or could be used for stationary storage is shown in Fig. 1d. Two important advantages of a KTFSI-based GDIB are the virtually unlimited natural abundance of its elemental constituents and the absence of toxic metals.

### DFT study of FSI^−^ anion intercalation into graphite

To better understand the staging mechanism of FSI^−^ anion intercalation into graphite, DFT calculations were performed. Complex anion species do not insert evenly into every graphite interlayer, but in a unique periodic manner called the staging mechanism as illustrated in Supplementary Fig. 2. Such a mechanism results from the balance between the van der Waals forces between the graphene layers on one side and the ionic repulsion between and within the intercalant layers^51^. This mechanism proceeds through different stages with a varying periodic repeat distance *I*_c_ (which is the distance between two neighboring FSI^−^ anion intercalant layers), and a stage-independent intercalant gallery height *d*_i_ (i.e. the distance between two neighboring graphite layers with intercalant FSI^−^ anions). A lower stage number (*n*) corresponds to a higher intercalant concentration and fewer empty graphene–graphene layers, so it also implies a higher charge storage capacity.

To simulate the staging mechanism, four different stages of intercalation are modelled (see Supplementary Fig. 3), and three different concentrations are considered for each stage. Considering the C_6_[FSI]_*x*_ formula unit, the *x*-values are 0.167, 0.334, and 0.50 for stage 1; 0.083, 0.167, and 0.250 for stage 2; 0.055, 0.112, and 0.167 for stage 3; and 0.042, 0.083, and 0.125 for stage 4. A 6 × 6 × 2 supercell of 288 carbon atoms is constructed for calculating stage 1 (Supplementary Fig. 4), stage 2 (Supplementary Fig. 5), and stage 4 (Supplementary Fig. 6). Another 6 × 6 × 3 supercell of 432 atoms was used for calculations of stage 3 (Supplementary Fig. 7).

**Fig. 2:**
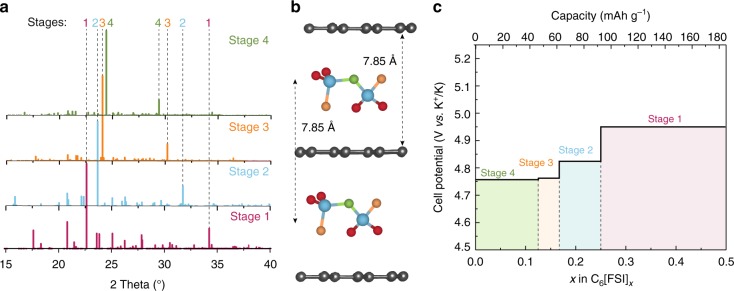
DFT calculations of FSI^−^ anion intercalation into graphite. **a** Simulated powder XRD patterns of charged graphite with intercalated FSI^−^ anions in different stages. The dashed lines represent the positions of the most intense peaks for stages 1, 2, 3, and 4. For stage 1, the peak at 2*θ* = 20.81° corresponds to the (*0k0*) plane, due to the lateral shift of graphite layers (AB stacked) upon FSI^−^ intercalation in the fully charged system. As there is much less shift of AB-stacking for other stages (stages 2, 3, and 4), we did not find any additional peaks for these systems (see Supplementary Fig. 8 and Note 3 for detailed explanation). **b** Schematic of FSI^−^ anion intercalated into graphite for stage 1 (*n* = 1). Fluorine, oxygen, sulfur, and nitrogen atoms representing FSI^−^ anion are shown in brown, red, blue, and green colors, respectively. **c** Calculated voltage profile of FSI^−^ anion intercalation into graphite as a function of the molar ratio between FSI^−^ and C (i.e., *x* in C_6_[FSI]_*x*_)

Next, we simulated the XRD patterns (Fig. 2a) based on the relaxed atomic coordinates obtained from the DFT calculations. For all the stages between *n* = 1 and 4, we found two dominant peaks indicated by dashed lines in Fig. 2a, which reflect the staging phenomenon. It is well-known that for stage n, the two most dominant XRD peaks correspond to the (*00n* + *1*) and (*00n* + *2*) planes, and the ratio *d*_00*n*+1_/*d*_00*n*+2_ reflects the stage number of graphite intercalation compound^52^. The periodic repeat distance is related to the d-spacing values as *I*_c_=(*n* + 1)*d*_00*n* + 1_=(*n* + 2)*d*_00*n*+2_=(*n* + m)*d*_00*n*+m_, where *d*_00*n*+1_, *d*_00*n*+2_, and *d*_00*n*+m_ are the *d*-spacings of (*00n* *+* *1*), (*00n* *+* *2*), and (*00n* *+* *m*) planes, respectively. For stage 1, the *d*_00*n*+1_ and *d*_00*n*+2_ spacing values are 3.92 and 2.61 Å, respectively, which result into a periodic repeat distance of *I*_c_ _=_ 2 × 3.92 = 3 × 2.61, ranging from 7.83 to 7.84 Å. Overall, these results indicate the formation of stage 1 FSI^−^ intercalated graphite in fully charged KFSI-graphite battery (Fig. 2b). In this case, the interlayer expansion is 134% upon the FSI^−^ anion intercalation, which is less than that for AlCl_4_ anion intercalation (150–160%)^53^ and comparable with the values of PF_6_^−^ and TFSI^−^ anion intercalation (130–140%)^54^. The fully intercalated stage 1 with the unit formula of C_6_[FSI]_0.5_ has a maximum storage capacity of 186 mAh g^–1^.

We calculated the voltage profile of KFSI-graphite DIB following the staging mechanism of FSI^−^ anion intercalation into graphite (Fig. 2c, see Supplementary Note 4 for details). It is clearly evident that the voltage increases with increasing extent of FSI^−^ anion intercalation. The calculated voltages for stages 4, 3, 2, and 1 are 4.75, 4.76, 4.82, and 4.95 V vs. K^+^/K, respectively, with their average value being 4.82 V vs. K^+^/K. Generally, besides the oxidative potential of graphite, there are other factors contributing towards the voltage of the anion intercalation into graphite. These are the anion desolvation energy (the energy cost to overcome in order to form separate anions from the electrolyte, the smaller is the anion—the higher is this energy) and intercalation energy (the energy cost to overcome to intercalate anion into graphite lattice, the larger is the anion—the higher is energy). Higher charging output is required to open up the interlayer spaces to insert larger anions. Higher desolvation and intercalation energies increase the intercalation voltage. For example, FSI^−^ intercalation voltage presented in this work is often found to be higher than PF_6_^−^ intercalation voltage^16,21,28^, which can be attributed to the larger anion size of FSI^−^ anion (van der Waal volume, 95 Å^3^), compared to PF_6_^−^ anion (van der Waal volume, 69 Å^3^)^55^. At the same time, smaller size of PF_6_^−^ anion would be expected to increase its solvation energy, somewhat neutralizing the lower intercalation barrier. Here then the solvent property comes into play and can shift this balance toward higher PF_6_^−^ intercalation voltage. For example, higher intercalation voltage of 5 V vs. Li^+^/Li has been reported for PF_6_^−^-based system when propylene carbonate was used as a solvent^56^, which has higher polarity than the of EC/DMC solvent mixture used in this work.

### In situ XRD probing of FSI^−^ intercalation

**Fig. 3:**
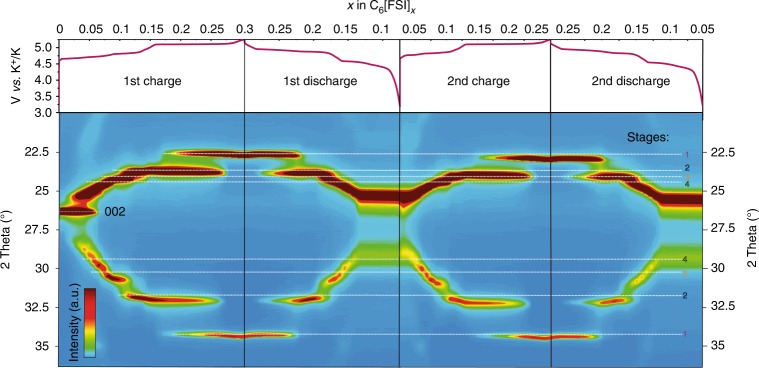
In situ XRD measurements of graphite during charging and discharging. Top: galvanostatic voltage profile measured at a current density of 10 mA g^−1^ for the first two cycles. Bottom: XRD patterns of graphite during cycling. The white horizontal dashed lines correspond to computed positions for different stages (shown in Fig. 2a)

Following the computational studies, the staging mechanism of FSI^−^ anion intercalation into graphite was experimentally probed by in situ XRD measurements. Figure 3 shows the obtained patterns during the charging and discharging of a graphite cathode during the first two cycles. The splitting of the (*002*) reflection of the pristine graphite upon charging evidences the intercalation process. The appearance of two dominant peaks corresponding to the (*00n* *+* *1*) and (*00n* + *2*) reflections also indicates the intercalation of FSI^−^ anions into graphite. After being fully charged at an upper potential limit of 5.25 V vs. K^+^/K, the positions of the two strongest peaks (22.76° and 34.40° in 2*θ*-units, or 0.39 and 0.26 nm in *d*-spacing units) match well with the computational results (white dash lines in Fig. 3), pointing to stage 1. From the galvanostatic curves, up to 0.3 moles of FSI^−^ anions can be intercalated into 6 moles of carbon, yielding a capacity of 112 mAh g^−1^. Some irreversible trapping of FSI^−^ anions occurs during the first charge, as shown by the capacity difference between the first charge and the first discharge. After the first discharge, the nearly identical capacities during the subsequent charge and discharge semicycles show the reversibility of the intercalation process. Irreversible trapping of FSI^−^ anions during first charge can be explained on the basis the irreversible changes occurred in graphite in terms of interlayer spacing (from 3.35 to 7.85 Å) as well as planes (from *(00l*) to (*0k0*)–(*00l*) plane, see Supplementary Fig. 9). This laterally shifted graphite structure gives FSI^−^ a stable plane to perfectly intercalate and interact with the neighboring graphitic layers with the maximum binding, leading to less favorable de-intercalation process and eventually trapping of some of the FSI^−^ anions inside the graphite structure as seen by the experimental observation of this study and some previous studies on PF_6_^−^ anions^57^. However, after the first charge/discharge cycle, the subsequent intercalation/deintercalation of FSI^−^ anions during charge/discharge processes becomes smooth due to already wetted (open spacious) graphite electrode structure^58^.

### Spectroscopic evidence for FSI^−^ intercalation

**Fig. 4:**
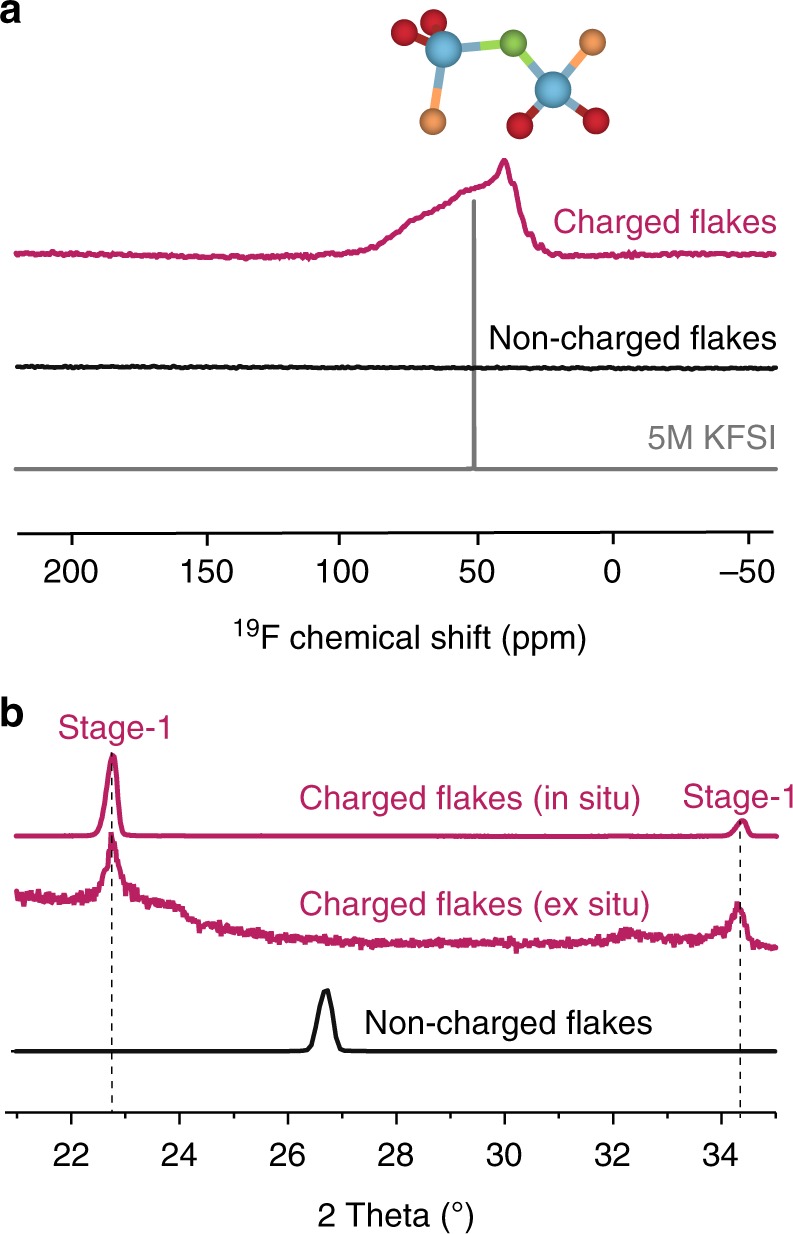
^19^F NMR and XRD characterization of graphite flakes. **a** ^19^F NMR spectra of charged and discharged graphite flakes (washed in EC/DMC), non-charged graphite flakes (reference), and 5 M KFSI/EC/DMC electrolyte. **b** XRD patterns of charged graphite, collected in situ and ex situ (i.e., after separation and washing of the graphite flakes). For comparison, the pattern for pristine graphite is also shown

The fate of FSI^−^ species was also examined with solution-state and solid-state ^19^F NMR spectroscopy (Fig. 4a). For these measurements, graphite was charged and discharged at a current density of 10 mA g^−1^ up to 5.25 V vs. K^+^/K, and then the excess electrolyte was rinsed off using pure EC/DMC solvent. The retention of the charged state during purification, handling, and measurements was confirmed by XRD measurements (Fig. 4b), where spectra collected in situ at the end of the electrochemical charging and ex situ after isolation and rinsing are compared. The ^19^F solid-state NMR spectrum of the charged and washed graphite exhibits a broad signal between 20 and 125 ppm, which is much weaker than the one observed for discharged graphite, confirming the intercalation of fluorine containing species upon charging . The similarity of the ^19^F frequency region of the signal in charged graphite with the isotropic chemical shift of KFSI electrolyte (measured by solution-state NMR) is an indication that one or several conformations of FSI^−^ are most likely the dominant form of intercalated F-containing species. The increased broadness of the the solid-state NMR spectrum is due to the impossibility to spin the sample due to the conductive properties of graphite. Nevertheless, the spectral shape of the static ^19^F solid-state spectra is reminiscent of a static axial tensor as one would expect by assuming that the isotropic FSI^−^ species, e.g., by rotation or tumbling of the ion, are surrounded by two flat and parallel graphene layers. A reference measurement was carried out by simply immersing graphite in the KFSI electrolyte without electrochemical intercalation and then washing it with EC/DMC. No signal was detected in this case.

### Electrochemical performance of KFSI-graphite DIB

**Fig. 5:**
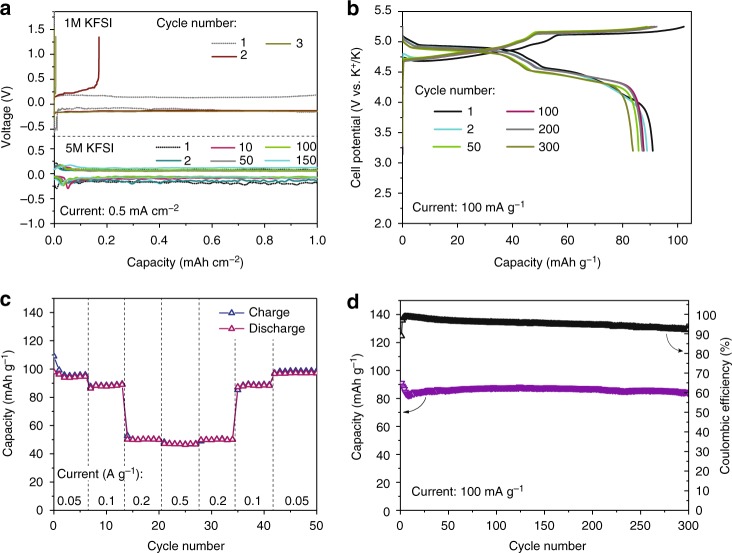
Electrochemical performance of KFSI-graphite DIB. **a** Galvanostatic plaiting (discharge) and stripping (charge) curves of coin-type cells composed of potassium metal as an counter and reference electrode and aluminum as an working electrode in 1 and 5 M KFSI/EC/DMC electrolyte, measured at areal current densities of 0.5 mA cm^−2^ (with areal capacity limitation of 1 mAh cm^−2^). **b**–**d** Galvanostatic charge–discharge voltage curves (**b**), rate capability measurements (**c**), and cycling performance of KFSI-graphite DIB (**d**)

First, the plating/stripping behavior of potassium in a 5 M KFSI/EC/DMC electrolyte (and also in 1 M KFSI/EC/DMC electrolyte for comparison) was evaluated using K|Al coin-type cells using Celgard 2400 separator. The cells were cycled at current density of 0.5 mA cm^−2^ (Fig. 5a). The charging and discharging processes were capacity-limited to a value of 1 mAh cm^−2^. In low concentrated electrolyte of 1 M, plaiting/stripping of potassium was not reversible with low Coulombic efficiency. On the contrary, stable cycling was measured in 5 M KFSI/EC/DMC electrolyte for at least about 150 cycles with 100% Coulombic efficiency. These results demonstrate the strong ability of 5 M KFSI-EC/DME electrolyte to sustain reversible plating/stripping of metallic potassium. In fact, these results and similar studies on electroplating of Li^59^, Na^60^, and K^61^ from highly concentrated electrolytes indicate that such electrolytes render a homogenizing cation flux that causes flat and uniform metal deposition^62^. We also tested another strategy for suppressing dendrite formation, by inserting a solid electrolyte barrier between the metal and liquid electrolyte. Employing potassium β″-alumina solid electrolyte to separate the cathode, we observed stable electroplating (Supplementary Figs. 10 and 11). Based on Supplementary Fig. 10, the potassium solid-state electrolyte did not impede the battery performance, sustaining the cathodic capacity of 80 mAh g^−1^ at 4.7 V vs. K^+^/K. Such solid-state design might be of interest for practical KFSI-GDIB batteries, e.g. those with molten and hence solvent-free KFSI electrolyte.

Galvanostatic cycling experiments of KFSI-graphite DIBs were performed employing custom-made TiN-coated stainless-steel coin-type cells. We note that common metals, such as Al or stainless steel can be easily corroded at high voltages above > 4.4 V vs. K^+^/K (4.5 V vs. Li^+^/Li). Thus, a TiN protective film was used to prevent oxidation of the cathode current collector upon cycling, in analogy to our earlier report on AlCl_3_-graphite battery^63^. The 500-nm-thick TiN film was grown by magnetron sputtering on one side of the stainless steel coin under nitrogen–argon atmosphere using a Ti target. The coin-type cell consisted of a thin film of potassium (used as a seed layer) pressed onto the aluminum foil, glass fiber separator soaked with 5 M KFSI/EC/DMC electrolyte, and graphite cathode. We note that, the highest capacity, average discharged voltage and initial Coulombic efficiency were observed for 5 M KFSI electrolyte indicating that it is the optimal concentration among different KFSI molarities in EC/DMC (Supplementary Fig. 12). The graphite cathode consisted of natural graphite flakes (loading: 10 mg cm^−2^) without using binders or conductive additives.

Figure 5b shows the galvanostatic voltage curves of KFSI-graphite DIB measured in the voltage interval of 3.2–5.25 V vs. K^+^/K at a current density of 100 mA g^−1^. The graphite cathodes showed very flat voltage profiles, with an initial Coulombic efficiency of 85%. The average charge and discharge voltages were 5.0 and 4.7 V vs. K^+^/K, respectively. Figure 5c shows the rate capability of KFSI-graphite DIB at different current densities of 50–500 mA g^−1^, yielding cathodic capacities of 98–47 mAh g^−1^, respectively. The drop of the capacity of KFSI-graphite DIB at high current densities of 200 and 500 mAh g^−1^ can be explained by the limiting effect of the ionic conductivity of KFSI/EC/DMC electrolyte at very high molarities (5 M, 65 wt% of KFSI)^64^. Importantly, when the current rate returned to 50 mA g^−1^ from 500 mA g^−1^, the capacity almost recovered fully. The corresponding power density at 500 mAh g^−1^ was as high as 1338 W kg^−1^, which compares favorably with batteries used in commercial grid-level storage, namely, lead acid (75–300 W kg^−1^) and vanadium redox-flow batteries (60–100 W kg^−1^). The cycling stability tests of graphite at a current density of 100 mA g^−1^ show a capacity retention in excess of 80 mA g^−1^ for at least 300 cycles with a Coulombic efficiency of 93–99%. In terms of energy efficiency, the obtained energy efficiency of 89% (derived from the ratio of the integrated areas of charged and discharge curves for 100th cycle (Fig. 5b), as an example) is comparable with that of commercial Li-ion batteries. We note that the lowering of the Coulombic efficiency is associated with oxidation/corrosion of edges of the custom-made TiN-coated stainless-steel coin-type cell (see Supplementary Fig. 13). TiN layer acts as a current collector, as presented in our earlier works for a range of battery chemistries^63^. On contrary, stainless steel is not suited for this kind of a battery due to its corrosion at such high voltages. Continuous coverage of the stainless-steel coin-type cell by the TiN conductive film was eventually limited by the magnetron sputtering technique used in this work, which enables to grow the TiN protective film only on relatively flat surfaces. These results suggest that future work should be focused on the further improved cell design of KFSI-graphite battery as well as other DIBs (which have similar problems) aiming to minimize oxidation/corrosion of the used current collectors. This scenario of corrosion is in good agreement with the observation of the high Coulombic efficiency initially and its decrease over time, due to formation, likely, of pinholes and other enhanced corrosion developments.

## Discussion

In summary, we present a KFSI-graphite DIB that employs a highly concentrated electrolyte (5 M KFSI in EC/DMC) to obtain high-energy densities up to 207 Wh kg^−1^. The corresponding cathodic capacity was up to 100 mAh g^−1^, and the average discharge voltage was 4.7 V. The staging mechanism of FSI^−^ anion intercalation into graphite was corroborated by in situ XRD data, DFT calculations, as well as solution-state and solid-state ^19^F NMR spectra. The KFSI-graphite DIBs exhibited high-energy efficiencies (ca. 89%), on par with well-established battery technologies, such as lead-acid batteries (90%), VRB (85%), and Li-ion batteries (90–95%). By combining a simple battery design with the use of abundant elements, GDIB appears as a strong contender among existing electrochemical cell concepts for grid-level batteries. Among other aspects, future work should focus on improving the battery design to support the large volumetric changes in the electrolyte and electrode during the intercalation process.

## Methods

### Chemicals and battery components

Natural graphite flakes (99.9%, ~10 mesh, Cat. no. 43319, Alfa Aesar), potassium bis(fluorosulfonyl) imide (KFSI, 99.9%, Suzhou Fluolyte Co., Ltd), ethylene carbonate (EC, battery grade, Novolyte), dimethyl carbonate (DMC, battery grade, Novolyte), potassium cubes (Merck), Al foil (MTI Corporation), and glass microfiber separator (GF/D, Cat no. 1823-257, Whatman) were used as received.

### Preparation of 5 M KFSI/EC/DMC electrolyte

The electrolyte was prepared by slowly mixing the KFSI white powder and EC/DMC solvent (1:1 v/v) in an argon-filled glove box. During the mixing, a highly isothermal reaction occurs and eventually a viscous transparent liquid is formed.

### TiN current collector fabrication

TiN was deposited on stainless steel (316L, Hohsen Corp.) substrates by pulsed DC magnetron sputtering using Ti target under an Ar/N_2_ atmosphere (Ar:N_2_ ratio of 82.5:23 in sccm) and pressure of 0.5 Pa. Before each deposition, both the substrate and target were pre-sputtered for 5 min in pure Ar. Then, the Ti target was poisoned under an 82.5:30 (sccm) for 5 min. The target power and temperature were set to 0.58 W cm^−2^ and 200 °C, respectively.

### Assembly and testing of the KFSI-graphite DIB

No binders or solvents were used to prepare the electrodes used in the coin-type cell batteries. Graphite was homogeneously distributed and pressed on the surface of TiN-coated stainless-steel cap, at the loading rate of 10 mg over ca. 1 cm^2^. The coin-type cells were assembled in an argon-filled glove box (O_2_ < 1 ppm, H_2_O < 1 ppm) using a glass fiber separator soaked with the 5 M KFSI/EC/DMC electrolyte (0.15 ml per coin-type cell). Thin potassium film pressed on the Al foil was used as both the reference and counter electrodes. These cells were cycled between 3.2 and 5.25 V on an MPG2 multichannel workstation (Bio-Logic). Cut-off potential of 5.25 V vs. K^+^/K was used to ensure high cyclic stability of KFSI-graphite DIB. As follows from cyclic voltammetry measurements of 5 M KFSI/EC/DMC electrolyte, its oxidation starts at higher voltages (Supplementary Fig. 14).

### Structural characterization of graphite

In situ and ex situ XRD measurements were performed using a Bruker AXS D8 Advance X-ray diffractometer with Ni-filtered (2 mm thickness) Cu Kα radiation (*λ* = 1.5406 Å) operated at 40 kV and 40 mA. The coin-type cell with TiN/polyimide window was used for in situ XRD measurements. For ex situ XRD studies, fully charged graphite flakes were removed from the cell inside the glove box and washed with EC/DMC solvent (1:1 v/v). Then, the graphite was sealed between scotch films and brought into air shortly prior to the XRD measurements.

### NMR spectroscopy measurements

Solid-state NMR spectra were acquired on a Bruker 9.4 T NMR spectrometer equipped with an Avance III console and a double resonance 2.5-mm solid state probe head. The proton channel could be tuned to ^19^F frequencies. Experiments were performed at room temperature without spinning the sample. To prepare the fully charged graphite flake samples, the flakes were removed from the cell inside a glove box and washed with EC/DMC solvent (1:1 v/v). Great care was taken so that the charged flakes did not contact any conducting materials including metals. Chemical shifts were referenced to CFCl_3_ (^19^F). The number of transients ranged from 256 to 1024 for ^19^F NMR spectra. One-pulse excitation sequences were used with pulse lengths of 5.75 μs. All spectra were acquired without decoupling, and the recycle delays were set to 5 s ^19^F respectively. The acquisition parameters for the ^19^F ssNMR spectra of graphite-containing samples are shown in Supplementary Table 2.

Solution-state NMR spectra were acquired on a Bruker 11.7 T solution-state NMR spectrometer equipped with a PABBO probe head and an Avance III console. Samples were filled into conventional 5-mm glass NMR tubes. Sealed glass capillaries containing C_6_D_6_ were added to the sample for locking to the deuterium signal of the deuterated benzene. The number of transients acquired was 256. Inverse gated H-1 pulse sequences were used with pulse lengths of 15.0 μs for ^19^F. During the acquisition of NMR spectra, a WALTZ16 decoupling sequence was performed using 80-μs proton decoupling pulses. The acquisition parameters for ^19^F solution-state NMR spectrum of 5 M KFSI are shown in Supplementary Table 3.

### DFT calculations

All the DFT calculations were carried using the Vienna Ab initio Simulation Package (VASP)^65^. Projected augmented wave (PAW) method^66,67^ was employed using an energy cut-off of 470 eV to describe the electronic wavefunctions. All the optimized structures were obtained by fully relaxing both atomic and lattice positions, until the Hellmann–Feynman forces on all atoms were smaller than 0.011 eV/Å. The Brillouin zone was sampled with Gamma-cantered *k*-point grid of 6 × 6 × 2 for unit cell and 2 × 2 × 1 for supercell calculations. All the systems were fully optimized, where the convergence criteria for total energy were set at 10^−5^ eV. The van der Waals interactions play a very crucial role in the layered systems. Therefore, we used van der Waals-corrected density functional theory (DFT-D3) proposed by Grimme to overcome the deficiencies of DFT in treating dispersion interactions^68^. The optimized interlayer spacing of pristine graphite (3.34 Å) is in close agreement with experimentally reported value (3.33 Å)^69^. The simulation results suggest that out of the two possible conformations of the FSI^−^ anion (Supplementary Fig. 15), the C2 (trans) conformer is more stable by 0.57 eV than the C1 (cis) conformer, which is very much in agreement with the previous calculations^70^. The FSI^−^ anion intercalates into graphite with horizontal orientation, leading to a metallic system (Supplementary Fig. 16) that could provide suitable electronic conductivity as a battery electrode.

## Electronic supplementary material

Supplementary Information

## Data Availability

The data that support the plots within this paper and other finding of this study are available from the corresponding author upon reasonable request.
