# Supplementary material for: High-energy-density dual-ion battery for stationary storage of electricity using concentrated potassium fluorosulfonylimide
Source: Nat Commun. 2018 Oct 26;9:4469. doi: 10.1038/s41467-018-06923-6 (PMC6203722; doi:10.1038/s41467-018-06923-6)
Supplement: Supplementary file 1 — Supplementary Information [file 41467_2018_6923_MOESM1_ESM.pdf]

Supporting Information for

High-energy-density dual-ion battery for stationary storage of electricity using concentrated potassium fluorosulfonylimide

Kostiantyn V. Kravchyk,[†,‡] *Preeti Bhauriyal,*[£] *Laura Piveteau,*[†,‡] *Christoph P. Guntlin,*[†,‡]

Biswarup Pathak, *[,£] *and Maksym V. Kovalenko,**[,†,‡]

Supplementary Figures

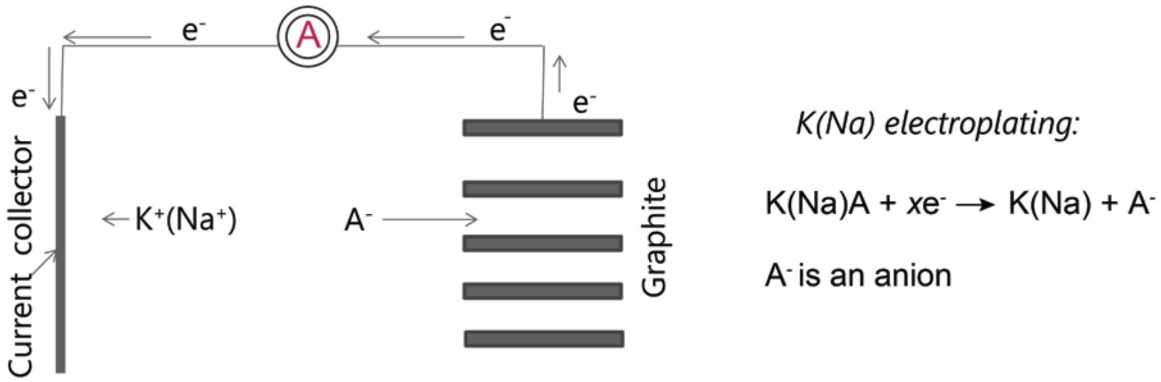

Supplementary Figure 1. Schematics of DIBs with graphite cathode utilizing electroplating reaction. Corresponding anodic electrochemical equation is shown on the right side of the figure.

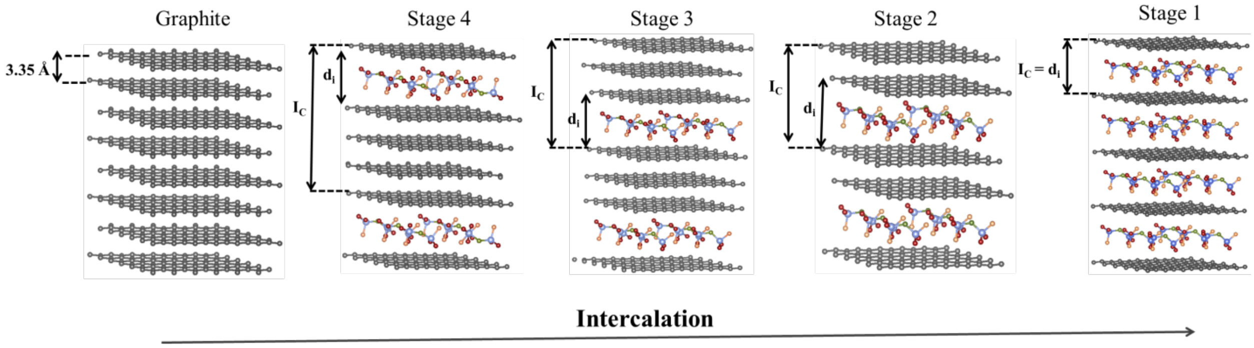

Supplementary Figure 2. Schematics of the staging mechanism of intercalation of FSI^{-} anions into graphite. Here, I_c = periodic repeat distance; d_i = intercalant gallery height.

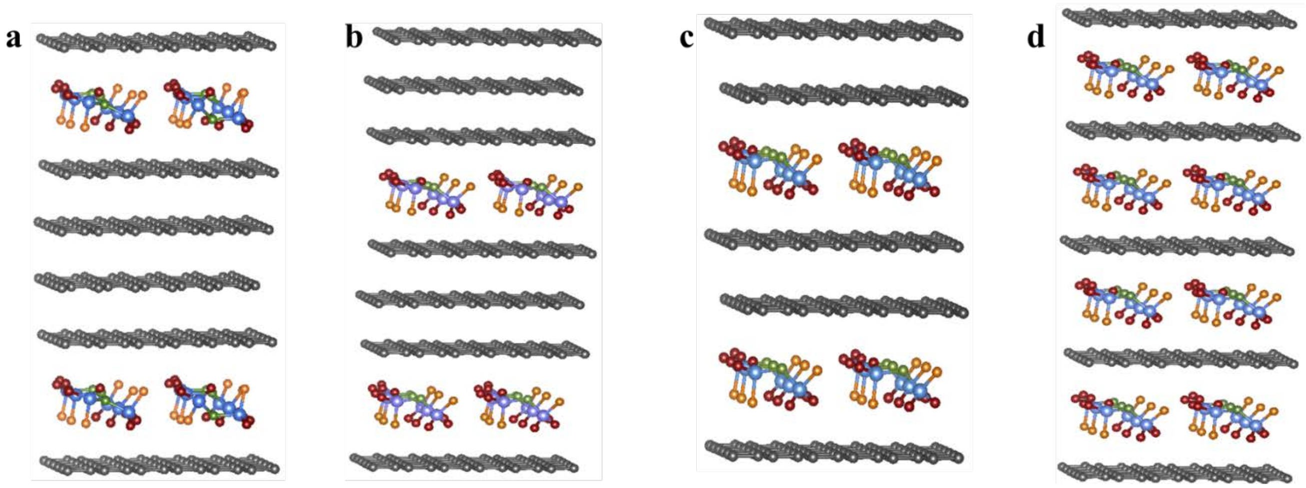

Supplementary Figure 3. Schematic representation (side view) of FSI^{-} anion intercalated into graphite for different intercalated stages. **a-d** Optimized structures of stage 4 (**a**), stage 3 (**b**), stage 2 (**c**) and, stage 1 (**d**).

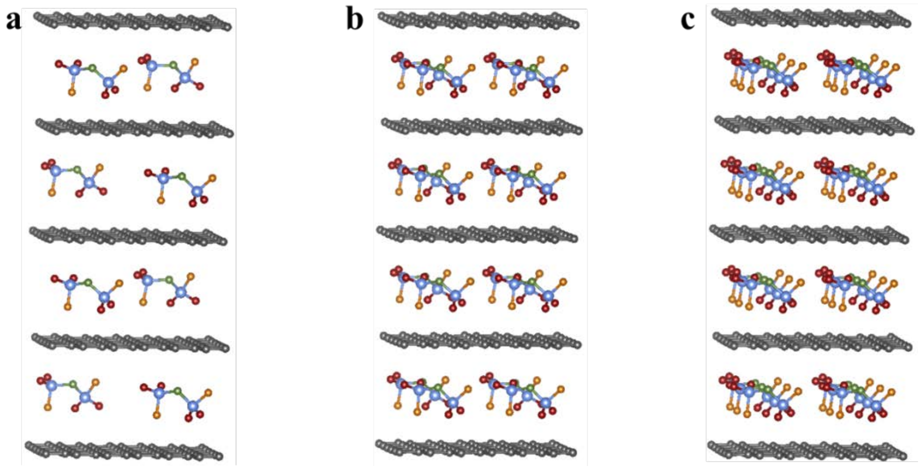

Supplementary Figure 4. Side view of 6×6×2 supercell of graphite for stage 1. **a-c** $C_6[FSI]_{0.0167}$ (**a**), $C_6[FSI]_{0.334}$ (**b**), and $C_6[FSI]_{0.5}$ (**c**) graphite/ FSI^{-} anion stoichiometries.

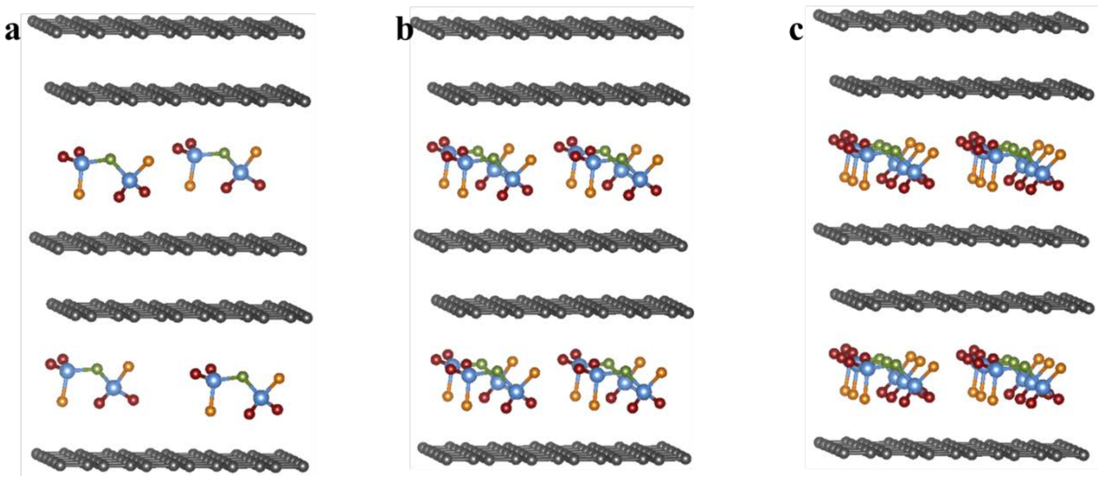

Supplementary Figure 5. Side view of 6×6×2 supercell of graphite for stage 2. **a-c** $C_6[FSI]_{0.083}$ (**a**), $C_6[FSI]_{0.167}$ (**b**), and $C_6[FSI]_{0.25}$ (**c**) graphite/ FSI^- anion stoichiometries.

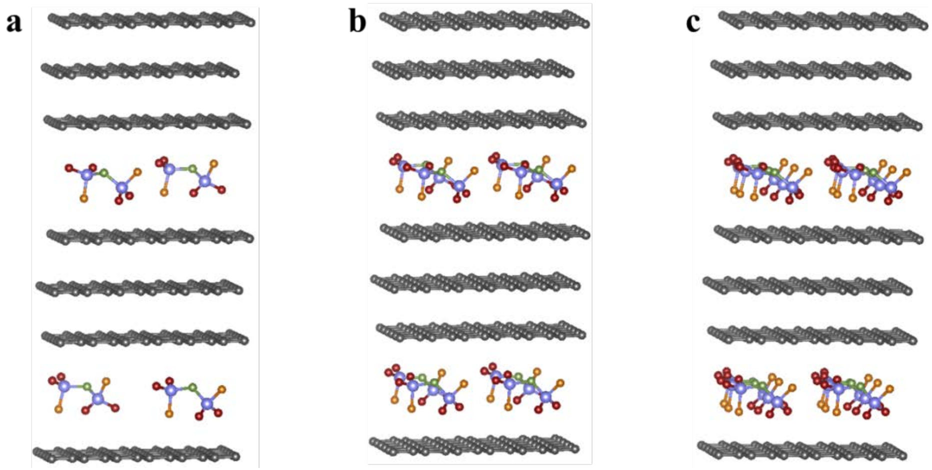

Supplementary Figure 6. Side view of 6×6×2 supercell of graphite for stage 3. **a-c** $C_6[FSI]_{0.055}$ (**a**), $C_6[FSI]_{0.112}$ (**b**), and $C_6[FSI]_{0.167}$ (**c**) graphite/ FSI^{-} anion stoichiometries.

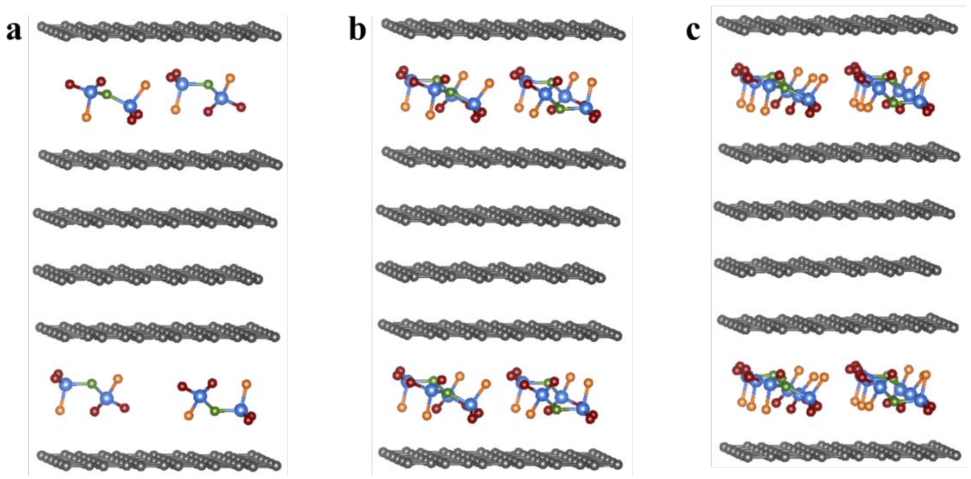

Supplementary Figure 7. Side view of 6×6×2 supercell of graphite for stage 4. **a-c** $C_6[FSI]_{0.042}$ (**a**), $C_6[FSI]_{0.083}$ (**b**), and $C_6[FSI]_{0.125}$ (**c**) graphite/ FSI^{-} anion stoichiometries.

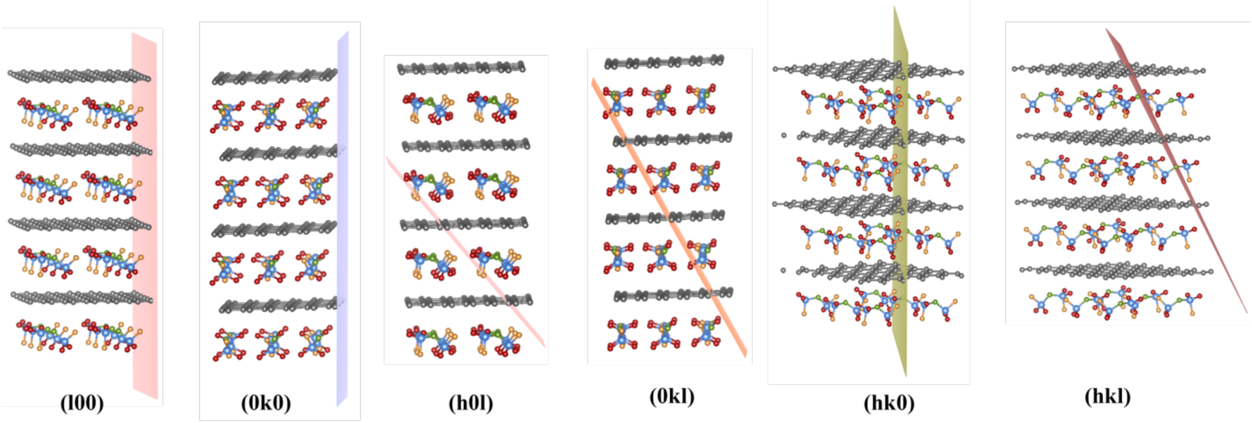

Supplementary Figure 8. Examples of planes in FSI^{-} intercalated graphite structures.

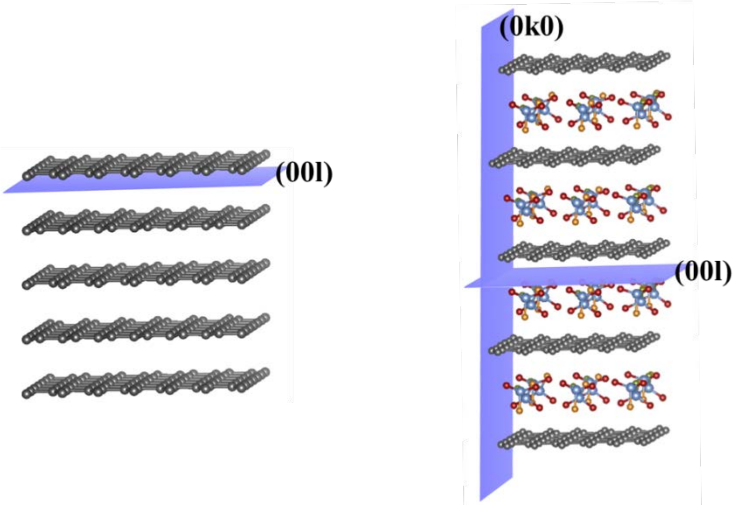

Supplementary Figure 9. Representation of pristine graphite and FSI^{-} anion intercalated graphite exhibiting (*00l*) plane, and (*00l*)-(*0k0*) planes, respectively.

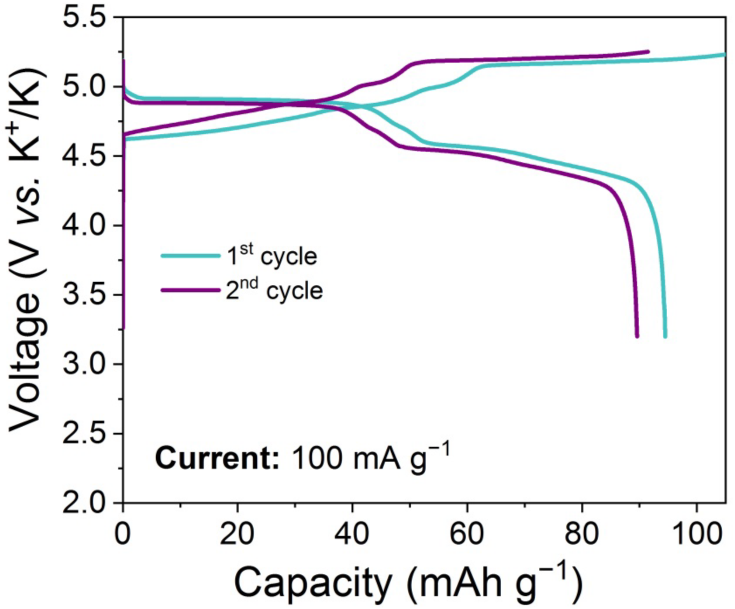

Supplementary Figure 10. Typical galvanostatic voltage profiles of KFSI-graphite DIB with potassium β″-alumina solid electrolyte measured at current density of 100 mA g^{-1}.

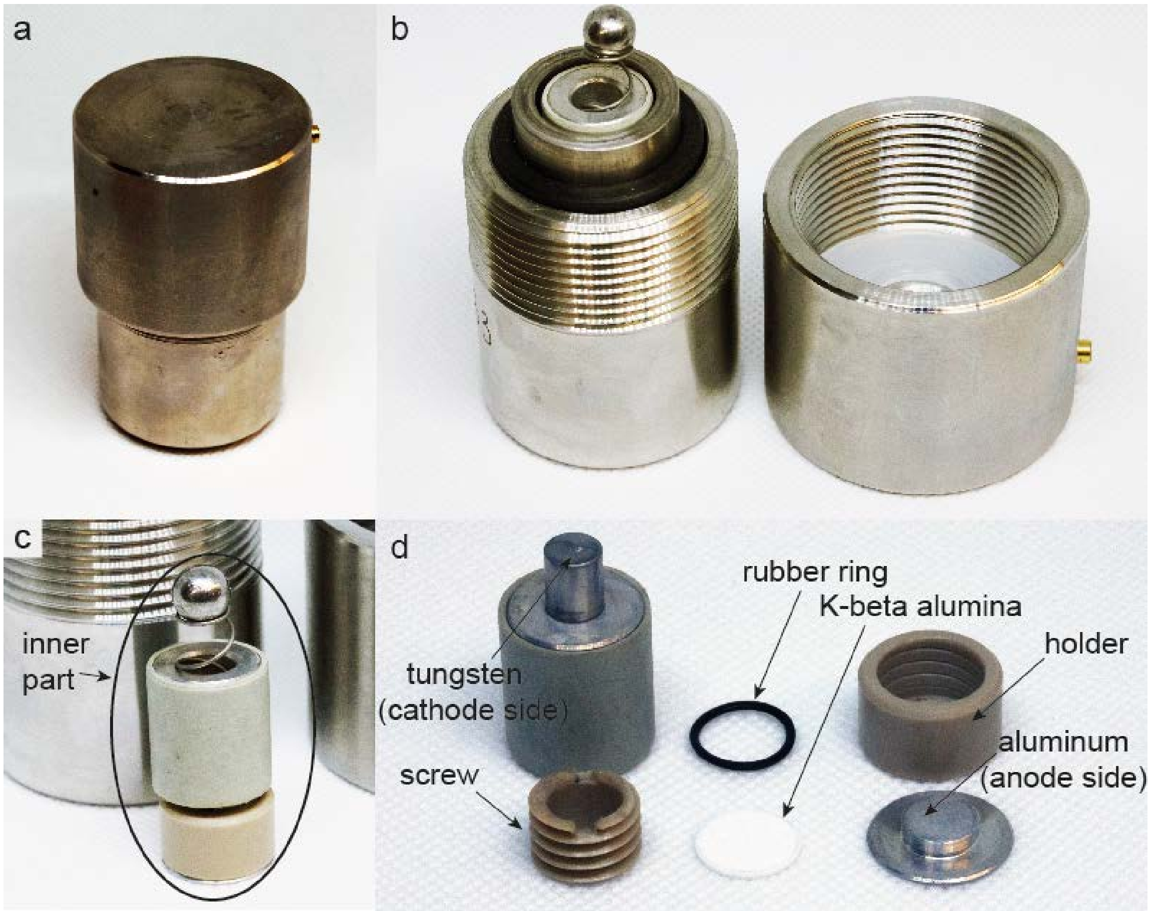

Supplementary Figure 11. Custom-made cell used for electrochemical measurements of KFSI-graphite DIB with potassium β″-alumina solid electrolyte.

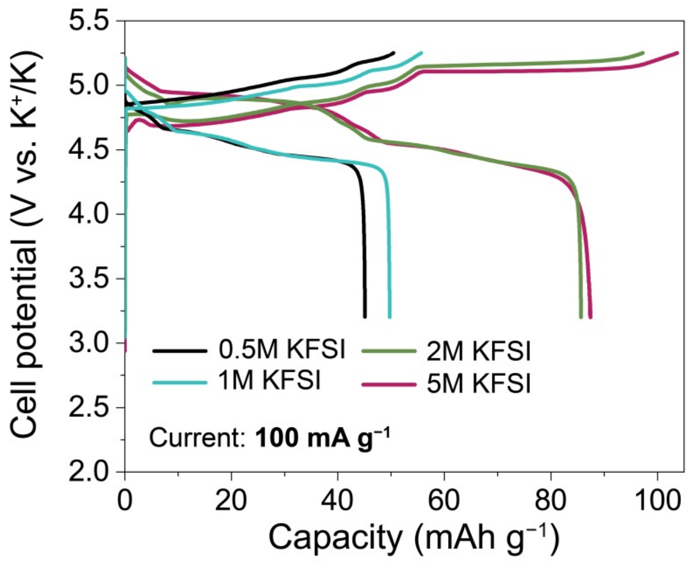

Supplementary Figure 12. Galvanostatic initial charge-discharge voltage curves of KFSI-graphite DIB with different KFSI molarities in EC/DMC.

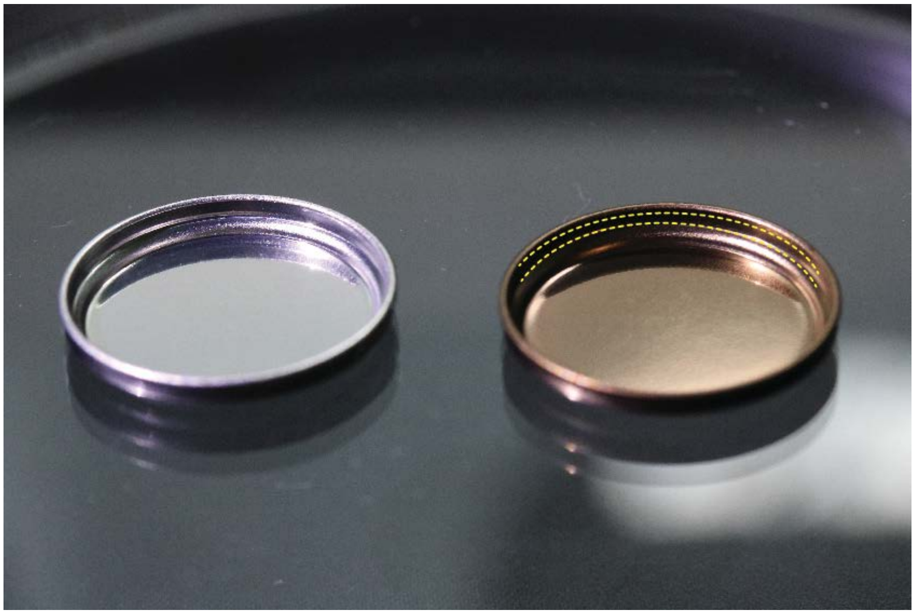

Supplementary Figure 13. Photograph of bare (left side) and TiN-coated (right side) stainless steel coin-type cells (bottom part). The edges of the custom-made TiN-coated stainless steel, where oxidation/corrosion of stainless steel takes place are indicated by yellow dash lines.

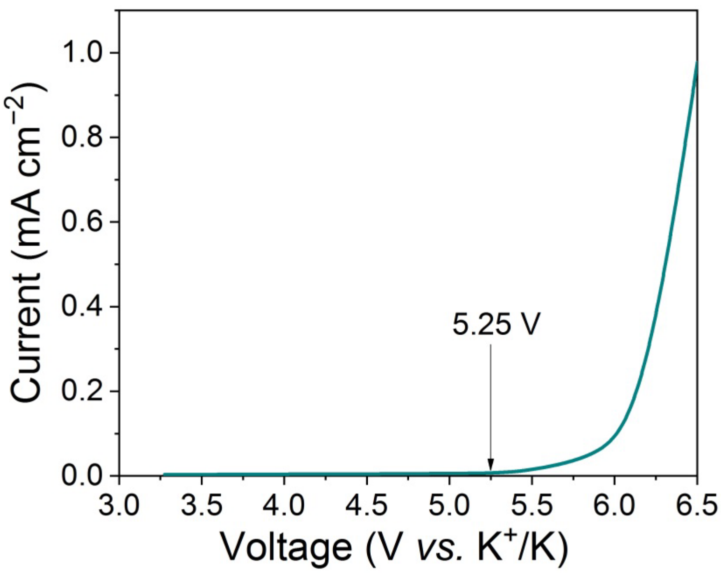

Supplementary Figure 14. Cyclic voltammetry of 5 M KFSI/EC/DMC electrolyte using tungsten current collector measured at a scan rate of 5 mV s^{-1}.

a

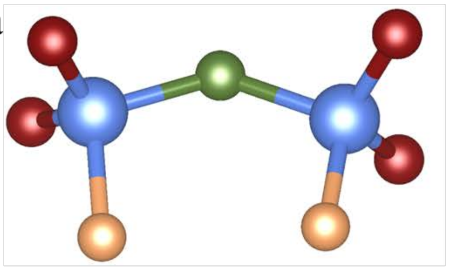

C1 (cis) conformer
S-O = 1.43
S-N = 1.60
S-F = 1.59
<F1-S1-N-S2 = 2.13°
<F2-S2-N-S1 = 16.91°

b

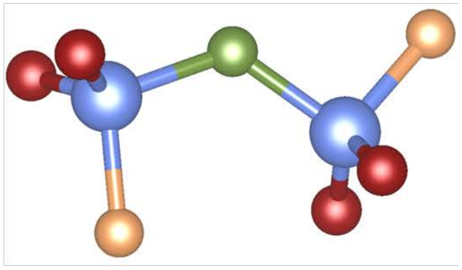

C2 (trans) conformer
S-O = 1.43
S-N = 1.61
S-F = 1.58 – 1.60
<F1-S1-N-S2 = 178.69°
<F2-S2-N-S1 = 3.78°

Supplementary Figure 15. Conformations of FSI^- anion. **a-b** C1 (cis) conformer (**a**), and C2 (trans) conformer (**b**). Oxygen, sulfur, nitrogen and fluorine atoms are shown in red, blue, green and brown colors, respectively.

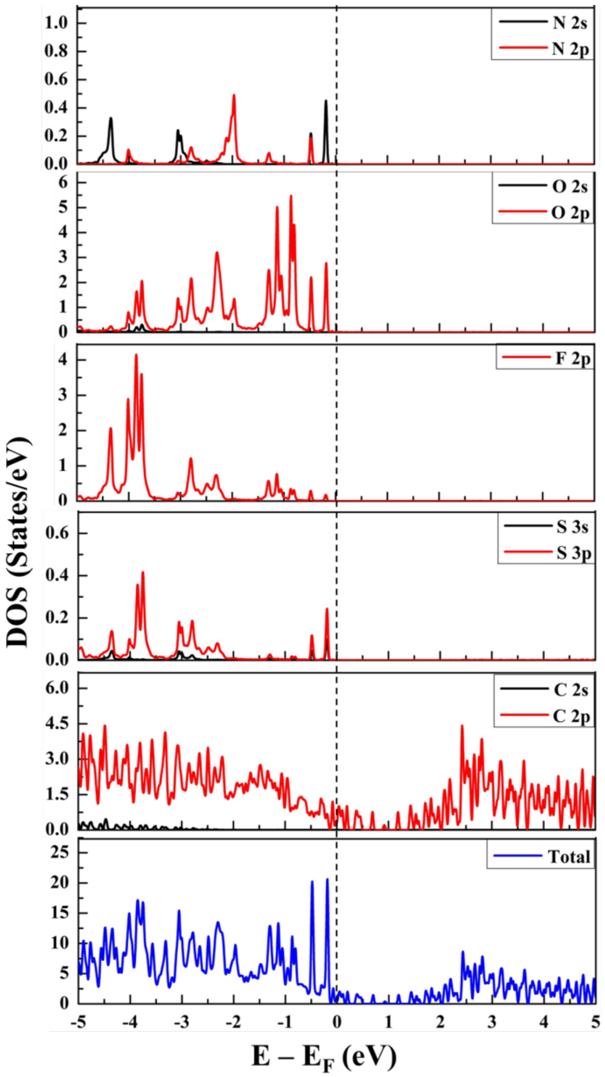

Supplementary Figure S16. Total DOS and projected DOS of FSI^- anion intercalated graphite, where the Fermi level is set at zero.

Supplementary Tables

Supplementary Table S1. Comparison of the theoretical gravimetric capacities and energy densities for various Li-free GDIBs. The gravimetric energy density of KFSI-graphite DIB utilizing electroplating reaction was calculated using Supplementary Eq. 8. The gravimetric energy density of other variants of Li-free GDIBs with intercalation/alloying reaction on the anode side were calculated using expression reported by Dahn *et al.*[1] The gravimetric energy density of $AlCl_3$-graphite was calculated using expression reported by Kravchyk *et. al.*[2]

**Battery**	**Anode**	**x**	**Cathode**	**Electrolyte/anolyte**	**Anode, mAh g^{-1}**	**Cathode capacity, mAh g^{-1}**	**Cell capacity, mAh g^{-1}**	**Cell voltage, V**	**Energy density, Wh kg^{-1}**
KFSI/graphite (present work)	KFSI	1	Graphite	5 M KFSI in EC/DMC	80	98	44	4.7	207
Pine needle derived carbon (PNC)/graphite[3]	PNC	0.09	Graphite	1 M $NaPF_6$ in EC/EMC	20	97	17	4.0	68
Carbon molecular sieve (CMS)/graphite[4]	CMS	0.09	Graphite	1 M $NaPF_6$ in EC/EMC	20	97	17	4.0	68
Sn/graphite[5]	Sn	1	Graphite	1 M $NaPF_6$ in EC/DMC/EMC	20	78	16	4.0	64
NaTFSI/graphite[6]	NaTFSI (Na plaiting)	1	Graphite	0.3 M NaTFSI in Pyr14TFSI	6	40	5	4.3	22
$NaPF_6$/freestanding meso-carbon microbead film	$NaPF_6$ (Na	1	FS-MCMB	1 M $NaPF_6$ in EC/DMC	20	84	16	4.1	66

(FS-MCMB)[7]	plaiting)								
Mesocarbon microbead/graphite[8]	Mesocarbon microbead	0.09	Graphite	1 M KPF_6 in EC/DMC/EMC	20	61	15	4.4	66
Sn/graphite[9]	Sn	2	Graphite	1 M KPF_6 in EC/DMC/EMC	20	60	15	4.2	63
Graphite/PTPAn[10]	Graphite	0.09	Polytriphe nylamine (PTPAn)	0.8 M KPF_6 in EC/DEC	16	60	13	2.9	38
Graphite/graphite[11]	Graphite	0.09	Graphite	0.8 M KPF_6 in EC/DMC	16	62	13	4	52
Graphite/graphite[12]	Graphite	0.09	graphite	0.3 M KTFSI in Pyr14TFSI	6	45	5	4.5	23
Soft carbon (SC)/graphite[13]	SC	0.08	graphite	1 M $NaPF_6$ in EC/DMC	19	80	15	3.6	54
$AlCl_3$/graphite[14]	$AlCl_3$ (Al plaiting)	0.75	graphite	$AlCl_3$/EMIMCl (2:1 molar ratio) or 3.4M $AlCl_3$ in $AlCl_3$/EMIMCl (1:1 molar ratio)	42	142	32	2	64

Abbreviations: EC = Ethylene carbonate; DMC = Dimethyl carbonate; EMC = Ethyl methyl carbonate; DEC = Diethyl carbonate; Pyr14TFSI = 1-Butyl-1-methylpyrrolidinium bis(trifluoromethanesulfonyl)imide; NaTFSI = Sodium(I) Bis(trifluoromethanesulfonyl)imide; KTFSI = Potassium(I) bis(trifluoromethanesulfonyl)imide; KFSI = Potassium bis(fluorosulfonyl)imide; EMIMCl = 1-Ethyl-3-methylimidazolium chloride.

Supplementary Table S2. Acquisition parameters for ^{19}F ssNMR spectra of graphite containing samples.

**Magnetic field**	9.4 T
**Temperature**	Room temperature
**Rotor diameter (mm)**	2.5
**Pulse Sequence**	one-pulse
**Number of scans**	1024
**Recycle delay (s)**	5
**Spectral width (kHz)**	938
**Spinning frequency (Hz)**	0
**Acquisition length (number of points)**	10240
**^{19}F 90° pulse width [π/2] (μs)**	5.75

Supplementary Table S3. Acquisition parameters for ^{19}F solution-state NMR spectrum of 5 M KFSI-EC/DMC.

**Magnetic field**	11.7 T
**Temperature**	Room temperature
**Rotor diameter (mm)**	2.5
**Pulse Sequence**	one-pulse
**Decoupling sequence**	Inverse gated H-1, WALTZ
**Number of scans**	256
**Recycle delay (s)**	8
**Spectral width (kHz)**	469
**Spinning frequency (Hz)**	0
**Acquisition length (number of points)**	11072

Supplementary Notes

Supplementary Note 1

Calculations of gravimetric cell-level capacity of GDIB utilizing electroplating reaction

GDIB utilizing electroplating/stripping reactions on the anode side is depicted schematically on the Figure S1. In this type of battery, KFSI salt (solved in solvent) acts as an anode being reduced on the current collector during charge of the battery.

Gravimetric cell level capacity of GDIB can be derived as follows:

$$Q_{cell} = Q_{anode} = Q_{cathode} \qquad (1)$$

where Q_{cell}, Q_{anode} and $Q_{cathode}$ are the charge of cell, anode and cathode.

Substituting Q by m and C (according to $Q = mC$) into Supplementary Eq. 1 one obtains:

$$C_{cell} = \frac{Q_{anode}}{m_a+m_c} = \frac{m_a C_a}{m_a+m_c} = \frac{m_a C_a}{m_a+\frac{m_a C_a}{C_c}} = \frac{C_a C_c}{C_a+C_c} \qquad (2)$$

or

$$C_{cell} = \frac{C_a C_c}{C_a+C_c} \qquad (3)$$

where C_c is the specific gravimetric capacity of the graphite cathode in mAh g^{-1} and C_a is the specific gravimetric capacity of the anode in mAh g^{-1}.

The capacity of anode in mAh g^{-1} can be calculated using the following equation:

$$C = \frac{xF}{M_{AM}} \qquad (4)$$

, where $F = 26.8 \times 10^3$ mAh mol^{-1} (Faraday constant), x is the charge of electroactive species and M_{AM} is molar mass of active material in g mol^{-1}.

Taking into account that our active material is KFSI salt solved in solvent, the capacity of anode should be calculated from the following equation:

$$C_a = \frac{xF}{M_{AM} + m_{solvent}} \qquad (5)$$

$M_{AM} + m_{solvent}$ part in Supplementary Eq. 5 can be expressed through the molarity (mol L^{-1}) of the electrolyte in discharged (M_d) and charged (M_c) state of battery and density of the electrolyte ρ in g ml^{-1}:

$$M_{AM} + m_{solvent} = \frac{\rho \cdot 10^3}{M_d - M_c} \qquad (6)$$

Therefore, the capacity of the anode can be written as follows:

$$C_a = \frac{xF(M_d - M_c)}{\rho \cdot 10^3} \qquad (7)$$

Substituting Supplementary Eq. 7 into Supplementary Eq. 4 one obtains

$$\text{Gravimetric } C_{cell} = \frac{Fx(M_d - M_c)C_c}{Fx(M_d - M_c) + C_c \rho \cdot 10^3} \qquad (\text{Ah kg}^{-1}) \qquad (8)$$

To estimate the gravimetric energy density, the C_{cell} value must be multiplied by the average battery voltage, $E = C_{cell} \cdot V$.

Supplementary Note 2

Calculations of volumetric cell-level capacity of GDIB utilizing electroplating reaction

Volumetric cell level capacity of GDIB can be derived as follows:

$$Q_{cell} = Q_{anode} = Q_{cathode} \tag{9}$$

where Q_{cell}, Q_{anode} and $Q_{cathode}$ are the charge of cell, anode and cathode.

Substituting Q by V and C (according to $Q = VC$) into Supplementary Eq. 9 one obtains:

$$C_{cell} = \frac{Q_{anode}}{V_a+V_c} = \frac{V_aC_a}{V_a+V_c} = \frac{V_aC_a}{V_a+\frac{V_aC_a}{C_c}} = \frac{C_aC_c}{C_a+C_c} \tag{10}$$

or

$$C_{cell} = \frac{C_aC_c}{C_a+C_c} \tag{11}$$

where C_c is the specific volumetric capacity of the graphite cathode in mAh ml^{-1} and C_a is the specific volumetric capacity of the anode in mAh ml^{-1}.

The capacity of anode in Ah L^{-1} can be calculated using the following equation:

$$C = \frac{xF}{V_{AM}} \tag{12}$$

where $F = 26.8 \times 10^3$ mAh mol^{-1} (Faraday constant), x is the charge of electroactive species and V_{AM} is volume of 1mol of active material in ml mol^{-1}.

Taking into account that our active material is KFSI salt solved in solvent, the capacity of anode should be calculated from the following equation:

$$C_a = \frac{xF}{V_{AM}+V_{solvent}} \tag{13}$$

$V_{AM} + V_{solvent}$ part in Supplementary Eq. 13 can be expressed through the molarity (mol L^{-1}) of the electrolyte in discharged (M_d) and charged (M_c) state of battery:

$$V_{AM} + V_{solvent} = \frac{10^3}{M_d - M_c} \qquad (14)$$

Therefore, the capacity of the anode can be written as follows:

$$C_a = \frac{xF(M_d - M_c)}{10^3} \qquad (15)$$

Substituting Supplementary Eq. 15 into Supplementary Eq. 12 one obtains

$$\text{Volumetric } C_{cell} = \frac{Fx(M_d - M_c)C_c\rho_c}{Fx(M_d - M_c) + C_c\rho_c \cdot 10^3} \quad (\text{Ah L}^{-1}) \qquad (16)$$

where ρ_c is density of bulk graphite in g ml^{-1}.

To estimate the volumetric energy density, the C_{cell} value must be multiplied by the average battery voltage, $E = C_{cell} \cdot V$.

Supplementary Note 3

Short explanation as to the appearance of additional peaks for stage 1

The charging process changes the structure of graphite in terms of interlayer spacing (from 3.35 Å to 7.85 Å) as well as planes. The FSI^- intercalated graphite structures possess additional planes such as (*h00*), (*0k0*), (*h0l*), (*0kl*), (*hk0*), and (*hkl*) other than (*00l*) planes, which are apparent in the XRD simulation plot as additional peaks. These planes appear as a consequence structural deformation of graphite on FSI^- intercalation such as the lateral shifting of graphite layers (AB stacked) upon FSI^- intercalation. These additional peaks are more intensive for stage 1 compared to other stages due to the more crowding of FSI^- anions causing larger structural changes, whereas, in higher stages, stage 2, stage 3, and stage 4, the deformation of graphite structure is less prominent leading to weakening of the peaks.

Supplementary Note 4

Voltage profile calculations of FSI⁻ anion intercalation into graphite

The voltage profile was calculated for KFSI-graphite dual ion battery (DIB) following the staging mechanism of FSI^- anion intercalation into graphite. It is well known that the electrolyte plays a very important role in the overall reaction mechanism of DIBs,[8-10] therefore its contribution is also included in the overall reaction and the corresponding voltage equation for theoretical calculations:

$$x\ KFSI(EC)_3DMC + C_n \leftrightarrow C_n(FSI)_x + x\ K + 3x\ (EC) + x\ DMC$$

$$V = \frac{(E_{C_n(FSI)_x} + xE_K + 3xE_{EC} + xE_{DMC}) - (E_{C_n} + xE_{KFSI(EC)_3DMC})}{xz} \quad (17)$$

where E_{C_n} and $E_{C_n(FSI)_x}$ are the energy of graphite before and after FSI^- anion intercalation, respectively; and x is the number of FSI^- anions intercalated in graphite. E_{EC}, E_{DMC}, and $E_{KFSI(EC)_3DMC}$ are the total energies of the respective solvents (ethylene carbonate, dimethyl carbonate, and $KFSI(EC)_3DMC$ electrolyte). E_K is the energy per atom of bulk potassium. E_{EC}, E_{DMC}, and $E_{KFSI(EC)_3DMC}$ are calculated by optimizing the EC, DMC, and $KFSI(EC)_3DM$ molecular species, due to the unavailability of their crystal structures.

Supplementary References

1. Dahn, J. R. & Seel, J. A. Energy and capacity projections for practical dual-graphite cells. *J. Electrochem. Soc.* **147**, 899–901 (2000).
2. Kravchyk, K. V., Wang, S., Piveteau, L. & Kovalenko, M. V. Efficient aluminum chloride–natural graphite battery. *Chem. Mater.* **29**, 4484–4492 (2017).
3. Wang, X., Zheng, C., Qi, L. & Wang, H. Carbon derived from pine needles as a Na^+-storage electrode material in dual-ion batteries. *Glob. Chall.* **1**, 1700055 (2017).
4. Wang, X., Qi, L. & Wang, H. Commercial carbon molecular sieves as a Na^+-storage anode material in dual-ion batteries. *J. Electrochem. Soc.* **164**, A3649–A3656 (2017).
5. Sheng, M., Zhang, F., Ji, B., Tong, X. & Tang, Y. A novel tin-graphite dual-ion battery based on sodium-ion electrolyte with high energy density. *Adv. Energy Mater.* **7**, 1601963 (2017).
6. Meister, P. et al. Sodium-based vs. lithium-based dual-ion cells: Electrochemical study of anion intercalation/de-intercalation into/from graphite and metal plating/dissolution behavior. *Electrochim. Acta* **228**, 18–27 (2017).
7. Liao, H.-J. et al. Freestanding cathode electrode design for high-performance sodium dual-ion battery. *J. Phys. Chem. C* **121**, 24463–24469 (2017).
8. Ji, B., Zhang, F., Wu, N. & Tang, Y. A dual-carbon battery based on potassium-ion electrolyte. *Adv. Energy Mater.* **7**, 1700920 (2017).
9. Ji, B., Zhang, F., Song, X. & Tang, Y. A novel potassium-ion-based dual-ion battery. *Adv. Mater.* **29**, 1700519 (2017).
10. Fan, L., Liu, Q., Xu, Z. & Lu, B. An organic cathode for potassium dual-ion full battery. *ACS Energy Lett.* **2**, 1614–1620 (2017).
11. Fan, L. et al. Potassium-based dual ion battery with dual-graphite electrode. *Small* **13**, 1701011 (2017).
12. Beltrop, K., Beuker, S., Heckmann, A., Winter, M. & Placke, T. Alternative electrochemical energy storage: potassium-based dual-graphite batteries. *Energy Environ. Sci.* **10**, 2090–2094 (2017).
13. Fan, L., Liu, Q., Chen, S., Xu, Z. & Lu, B. Soft carbon as anode for high-performance sodium-based dual ion full battery. *Adv. Energy Mater.* **7**, 1602778 (2017).
14. Wang, S., Kravchyk, K. V., Krumeich, F. & Kovalenko, M. V. Kish graphite flakes as a cathode material for an aluminum chloride–graphite battery. *ACS Appl. Mater. Interfaces* **9**, 28478–28485 (2017).
